# Neuronatin regulates whole‐body metabolism: is thermogenesis involved?

**DOI:** 10.1096/fba.2020-00052

**Published:** 2020-09-02

**Authors:** Jessica L. Braun, Mia S. Geromella, Sophie I. Hamstra, Val A. Fajardo

**Affiliations:** ^1^ Department of Kinesiology Brock University St. Catharines ON USA; ^2^ Centre for Bone and Muscle Health Brock University St. Catharines ON USA; ^3^ Centre for Neuroscience Brock University St. Catharines ON USA

**Keywords:** brown adipocyte, calcium, insulin, leptin, SERCA

## Abstract

Neuronatin (NNAT) was originally discovered in 1995 and labeled as a brain developmental gene due to its abundant expression in developing brains. Over the past 25 years, researchers have uncovered NNAT in other tissues; notably, the hypothalamus, pancreatic β‐cells, and adipocytes. Recent evidence in these tissues indicates that NNAT plays a significant role in metabolism whereby it regulates food intake, insulin secretion, and adipocyte differentiation. Furthermore, genetic deletion of *Nnat* in mice lowers whole‐body energy expenditure and increases susceptibility to diet‐induced obesity and glucose intolerance; however, the underlying cellular mechanisms remain unknown. Based on its sequence homology with phospholamban, NNAT has a purported role in regulating the sarco(endo)plasmic reticulum Ca^2+^ ATPase (SERCA) pump. However, NNAT also shares sequence homology with sarcolipin, which has the unique property of uncoupling the SERCA pump, increasing whole‐body energy expenditure and thus promoting adaptive thermogenesis in states of caloric excess or cold exposure. Thus, in this article, we discuss the accumulating evidence suggestive of NNAT’s role in whole‐body metabolic regulation, while highlighting its potential to mediate adaptive thermogenesis via SERCA uncoupling.

**Abbreviations**
BAT: brown adipose tissue
BMR: basal metabolic rate
EDL: extensor digitorum longus
NNAT: neuronatin
PLN: phospholamban
SERCA: sarco(endo)plasmic reticulum Ca^2+^ ATPase
SLN: sarcolipin
SNP: single‐nucleotide polymorphism
SR/ER: sarco(endo)plasmic reticulum
UCP‐1: uncoupling protein 1
WAT: white adipose tissue

## 1 INTRODUCTION

The neuronatin (*NNAT*, 3.9 kb) gene is paternally inherited with the maternal allele silenced via DNA methylation.^1^, ^2^ Genomic imprinting such as this results in monoallelic expression—a characteristic of several other genes critical for growth, development, and metabolism.^3^ Through alternative splicing, *NNAT* mRNA produces two isoforms, NNATα (81 amino acids) and NNATβ (54 amino acids),^4^, ^5^ though the extent with which these isoforms are functionally redundant or divergent remain to be uncovered. *NNAT*/*Nnat* mRNA is enriched in the developing brain, and while downregulated thereafter, its expression is maintained throughout adulthood.^6^ During brain development, NNAT has been shown to generate critical intracellular Ca^2+^ ([Ca^2+^]_i_) signals important for differentiation of stem cells, synaptic formation, and plasticity.^7^, ^8^, ^9^ Underlying its involvement in cellular Ca^2+^ regulation, NNAT has putative role in binding to and inhibiting the sarco(endo)plasmic reticulum Ca^2+^ ATPase (SERCA) that actively transports Ca^2+^ into the sarco(endo)plasmic reticulum (SR/ER). This is based on ~50% amino acid sequence homology with phospholamban (PLN), a well‐known SERCA inhibitor found in cardiac and skeletal muscles.^10^, ^11^, ^12^ More recently and aside from brain development, NNAT has been detected and its role investigated in pancreatic β‐cells,^13^ adipocytes,^14^ and the hypothalamus^15^ with one prevailing theme—metabolism. In this article, we highlight the role of NNAT in whole‐body metabolism specifically with glucose homeostasis, appetite regulation, adipocyte function, and energy expenditure. In the latter, we discuss the potential for NNAT to act as a mediator of adaptive thermogenesis via SERCA‐mediated Ca^2+^ futile cycling.

## 2 GLUCOSE HOMEOSTASIS

Proper blood glucose homeostasis is essential for a healthy life and prolonged hyperglycemia can increase risk of diabetes mellitus, cardiovascular disease, and kidney disease.^16^, ^17^, ^18^ Pancreatic β‐cells sense changes in blood glucose levels and, under conditions of elevated circulating glucose, release insulin via Ca^2+^‐dependent exocytosis.^19^ NNAT was first discovered in β‐cells in 1997,^13^ however, much of what we know on its physiological role has only recently surfaced. In 2005, Chu & Tsai^20^ found that silencing NNAT with siRNA in β‐cells resulted in impaired insulin secretion in response to elevated glucose. Later, both NNATα and NNATβ expressions were found in the ER, where its expression and localization increased with elevated glucose concentration.^21^ Furthermore, Joe et al.,^21^ showed that both NNAT isoforms can in effect increase [Ca^2+^]_i_, in β‐cells, which not only supports NNAT’s role in regulating SERCA but also provides the cellular mechanism in which NNAT promotes insulin secretion (through Ca^2+^‐mediated exocytosis).

**FIGURE 1:**
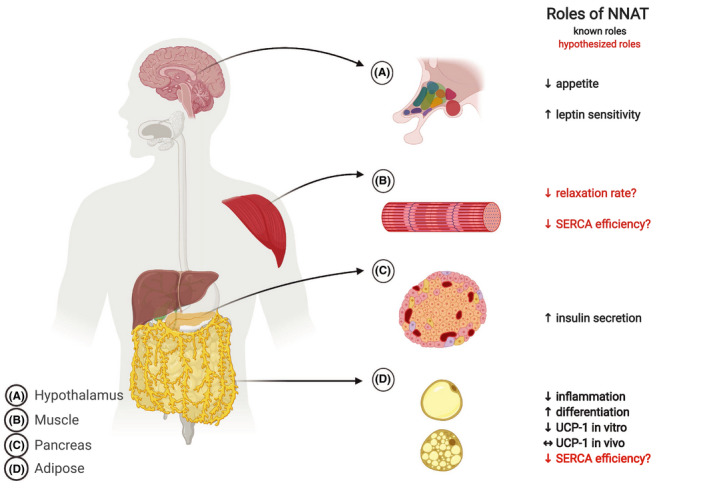
Schematic illustration of NNAT’s known and hypothetical roles in regulating metabolism across several tissues throughout the body. A, NNAT acts in the hypothalamus to suppress appetite by altering leptin sensitivity. B, In muscle, we hypothesize that NNAT regulates the SERCA pump by reducing its coupling ratio (Ca^2+^ transport efficiency) thereby mediating muscle‐based thermogenesis. C, In pancreatic β‐cells, NNAT has an active role in regulating insulin secretion by regulating [Ca^2+^]_I_ levels. D, NNAT is a white adipose tissue marker where it stimulates differentiation and adipogenesis. In vitro, NNAT represses the browning program; however, this effect has not been observed in vivo. We hypothesize that NNAT uncoupling of SERCA may also provide a UCP‐1 independent mechanism for thermogenesis in brown/beige adipocytes

In 2018, Millership et al. set out to provide the first in vivo evidence of NNAT participating in glucose homeostasis using both global NNAT knockout (NNAT^KO^) and β‐cell specific NNAT^KO^ C57BL/6J mice.^22^ Similar to the in vitro experiments from previous studies, NNAT expression was found to be elevated with higher circulating glucose. Also coherent with the previous in vitro work, NNAT^KO^ and β‐cell specific NNAT^KO^ mice displayed drastic impairments in glucose‐stimulated insulin secretion.^22^ When exposed to a high‐fat Western diet, the β‐cell NNAT^KO^ mice had greater elevations in fasting blood glucose levels and worsened glucose tolerance.^22^ Altogether, evidence from these studies clearly demonstrates a role for NNAT in maintaining glucose homeostasis by regulating pancreatic insulin secretion (Figure 1).

## 3 ADIPOCYTE DIFFERENTIATION AND FUNCTION

There are two main types of adipose tissue in the body: white adipose tissue (WAT) and brown adipose tissue (BAT). WAT is large unilocular cells that store excess energy in the form of triglycerides and release fatty acids when required. Conversely, BAT is multilocular cell with an abundance of mitochondria and functions primarily as an energy‐dissipating organelle critical for thermoregulation.^23^ BAT contains uncoupling protein 1 (UCP‐1) in the mitochondria where it acts to dissipate the proton gradient (generated by oxidative phosphorylation) uncoupling it from ATP synthesis and instead releasing the free energy as heat.^23^, ^24^ This process drives the combustion of fatty acids and glucose to generate the high‐energy intermediates that enter the electron transport chain to restore the proton gradient. A third type of adipocyte, beige, or “brite” (brown‐in‐white), has intermediate morphology with WAT and BAT. Like BAT, beige adipose also contain an abundance of mitochondria positive for UCP‐1.^23^ Thus, both BAT and beige adipocytes are important mediators of adipose‐based thermogenesis.

In 2005, NNAT was found in adipocytes with the investigation of the gene expression profiles of Zucker diabetic fatty rats.^25^ NNAT expression appears to vary with body composition. For example, NNAT was significantly increased in 6‐week‐old diabetic fatty mice compared to their lean counterparts,^25^ whereas, in lipodystrophic mice, there was a reduction in NNAT.^26^ Finally, high NNAT mRNA was found in adult adipocytes of obese rats.^27^ Collectively, this has led to the notion that NNAT is a WAT marker. Indeed, cold exposure which causes browning in subcutaneous WAT, has been shown to reduce NNAT mRNA expression.^28^ Conversely, warming mice leads to brown and beige adipose tissue “whitening” with a concomitant increase in NNAT mRNA.^29^ Moreover, NNAT may not only be associated with the WAT phenotype but may also play an active role as studies in 3T3‐L1 adipocytes have shown that NNAT stimulates adipogenesis through increasing [Ca^2+^]_I_,^27^ which is consistent with NNAT’s putative role as a SERCA regulator. Finally, silencing NNAT in cultured adipocytes has been shown to induce a brown‐like phenotype with increased expression of UCP‐1.^14^

Based on the findings that show that NNAT plays a role in repressing browning and increasing adipogenesis, one could hypothesize that NNAT would contribute to diet‐induced obesity. However, Millership et al.^30^ recently showed that NNAT knockout mice were more susceptible to diet‐induced obesity and glucose intolerance, and that contrary to previous in vitro work, *Nnat* deletion in mice did not induce a browning phenotype since there were no changes in UCP‐1 expression. Interestingly, these results are in line with recent studies in humans showing that NNAT *mRNA* is reduced in the obese children.^14^ Furthermore, SNPs have been linked to human obesity.^31^ Therefore, though the exact role of NNAT in adipose tissue remains unclear, it is evident both in rodents and in humans that NNAT deletion and mutations are linked to obesity. Obesity is associated with chronic low‐grade inflammation particularly with heightened adipose inflammation and resistance to insulin.^32^ With increased inflammation, free fatty acids diffuse out of the cells and can be taken up by other tissues and organs, leading to similar effects and the potential development of type 2 diabetes.^32^ Recently, NNAT has been shown to participate in an anti‐inflammatory pathway in adipocytes as well as promoting the effects of adiponectin.^33^ Adiponectin stimulates fatty acid oxidation and additionally serves as an anti‐inflammatory agent,^34^ providing a potential mechanism behind losses or mutations of NNAT being associated with obesity and its co‐morbidities.

## 4 APPETITE REGULATION

Though typically classified as a fat storer, WAT is not a metabolically inert tissue and is able to secrete hormones that aid in metabolic regulation. Leptin is a hormone released by WAT and its main role is to decrease food intake by binding to leptin receptors in the hypothalamus.^23^, ^30^ In addition to WAT, NNAT is expressed in the hypothalamus where its expression is thought to be positively regulated by leptin.^35^ In response to 24‐hour fasting, when leptin levels are lowered, hypothalamic *Nnat* mRNA was reduced in both lean and obese rats.^15^, ^31^ However, there are discrepant findings in high‐fat fed mice where plasma leptin levels were significantly increased but no changes in hypothalamic *Nnat* expression were observed.^15^ Furthermore, mice on a calorie‐restricted diet again showed no changes in NNAT expression in the hypothalamus nor any correlation between leptin levels.^15^ In any case, the hypothalamic expression of NNAT seems to be important for leptin signaling, and *Nnat* ^KO^ mice fed a high‐fat diet displayed reduced leptin sensitivity and hyperphagia, all of which would contribute to the enhanced weight gain previously observed.^30^ Finally, on the opposite end of the spectrum, variants in the NNAT gene and its subsequent expression are associated with individuals diagnosed with anorexia nervosa.^36^ Thus, accumulating evidence suggests that NNAT may repress appetite by ensuring leptin sensitivity though the exact cellular mechanisms are currently unknown (Figure 1).

## 5 ENERGY EXPENDITURE AND IMBALANCE

At the cellular level, obesity is caused by an energy imbalance whereby caloric intake exceeds energy expenditure. While the fact that *Nnat* ^KO^ mice fed a high‐fat diet gained more weight compared with their wild‐type littermates can be partly attributed to reduced leptin sensitivity and increased food intake, these mice also displayed significant reductions in whole‐body energy expenditure.^30^ Thus, in the absence of *Nnat*, mice are largely exposed to an energy imbalance the predisposes them to diet‐induced obesity that along with impaired insulin secretion would contribute to the glucose intolerance. Furthermore, the lowered daily energy expenditure occurred during both active (dark) and inactive (light) periods. The *Nnat* ^KO^ mice exhibited lower physical activity counts compared with wild type, which would contribute to the reduced energy expenditure during the dark periods.^30^ However, the lowered energy expenditure particularly at rest has been left unexplained.

## 6 SERCA UNCOUPLING: A ROLE FOR NNAT?

Basal metabolic rate (BMR) represents a collective measurement of all cellular activity in a resting state and contributes to 60%‐75% of total daily energy expenditure.^37^ Skeletal muscle comprises 40%‐50% of the mammalian body.^38^ Due to its highly energetically active nature and abundance in the body, skeletal muscle accounts for 20%‐25% of total BMR.^39^ The main contributor to energy expenditure in skeletal muscle are the SERCA pumps, which account for nearly 50% of resting metabolic rate in skeletal muscle.^39^ Due to its significant contribution to resting metabolic rate in skeletal muscle, as well as its large contribution to energy expenditure during active states, SERCA has become a viable therapeutic target for combatting energy imbalance disorders such as obesity. Based on its binding capacity the SERCA pump has an optimal coupling ratio of 2 Ca^2+^ transported into the SR for every 1 ATP hydrolyzed.^40^, ^41^, ^42^ However, reducing SERCA’s coupling ratio creates a futile Ca^2+^ cycle, where more ATP is required to transport Ca^2+^ into the SR. Studies aimed at lowering SERCA’s coupling ratio have all shown therapeutic promise in the fight against diet‐induced obesity and glucose intolerance.^43^, ^44^, ^45^, ^46^

**FIGURE 2:**
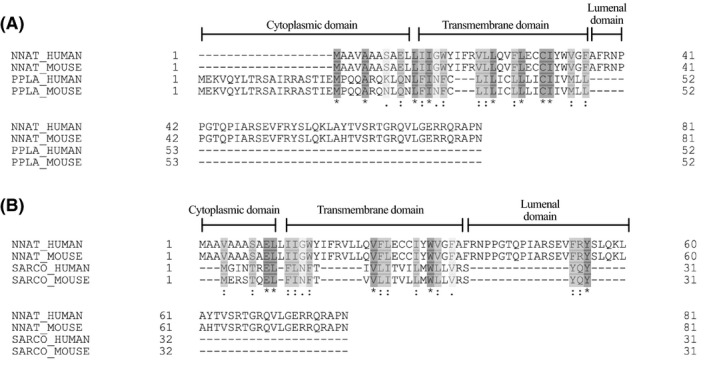
NNAT is homologous with SLN and PLN. UniProt sequence alignment between human and mouse *Sln* and *Nnat* (A) as well as *Pln* and *Nnat* (B). Cytoplasmic, transmembrane, and luminal domains of NNAT and SLN are shown in each figure. Increasing shades of gray indicate increasing in similarities with the darkest intensity indicative of identical amino acids (*). Sequence alignment of NNAT with PLN and SLN show the majority of homology in the transmembrane domain—the domain that interacts with SERCA and interferes with Ca^2+^ binding

NNAT shares 50% sequence homology with the allosteric SERCA regulator, PLN (Figure 2A).^27^ PLN acts by binding to the SERCA transmembrane domain causing conformational changes that decrease SERCA’s affinity for Ca^2+^.^11^ In muscle, PLN inhibition of SERCA prolongs relaxation and reduces contractility via a reduction in SR Ca^2+^ stores. Overexpression of PLN and increases in the ratio of PLN relative to SERCA has been implicated in dilated cardiomyopathy, heart failure, and skeletal muscle myopathies.^11^, ^47^, ^48^, ^49^ Thus, finding ways to reduce the amount of PLN relative to SERCA is an active field of research.^50^, ^51^

**FIGURE 3:**
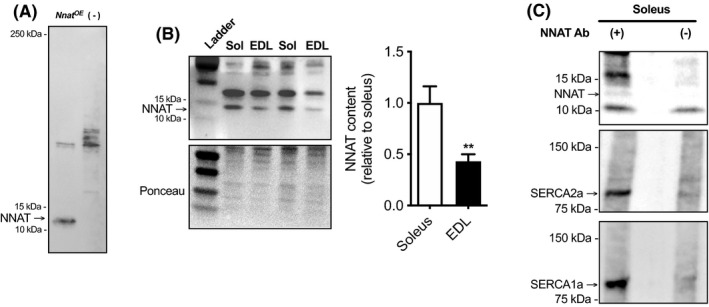
NNAT is expressed in murine skeletal muscle and binds to SERCA2a and SERCA1a. A, An NNAT overexpression cell lysate (NBP2‐04902, Proteintech) shows NNAT resolves at ~13 kDa. An empty vector was also included as a negative control. B, Representative Western blot images of NNAT protein expression in soleus and extensor digitorum longus (EDL) muscles and densitometric analyses of NNAT content in soleus and EDL muscles. Values were normalized to total protein and are presented relative to soleus. C, Co‐immunoprecipitation was performed with NNAT antibody (Ab) pull‐downs in type soleus muscle homogenate (n = 3). The eluent shows positive detection of NNAT, SERCA2a, and SECA1a. A negative control that contained only muscle homogenates and protein G magnetic beads without NNAT Ab shows minimal SERCA1a and SERCA2a binding to the protein G beads. **P ≤ .01, using a Student's unpaired *t* test, (n = 6 per group). For B and C, experiments were performed on 3‐4‐mo‐old female BL6;129 mice (Jackson Laboratories). Mice were fed a standard chow diet and housed under 12 h light:dark periods; and all animal protocols were approved by the Brock University Animal Care Committee. Western blotting and co‐immunoprecipitation protocols were performed as previously described using 4%‐15% TGX precast gels (Bio‐Rad Laboratories) and PVDF membranes.^50^, ^60^, ^61^, ^62^ Primary antibodies for NNAT were obtained from ProteinTech (26905‐1‐AP), whereas SERCA1a (MA3‐912) and SERCA2a (MA3‐919) were obtained from ThermoFisher

**FIGURE 4:**
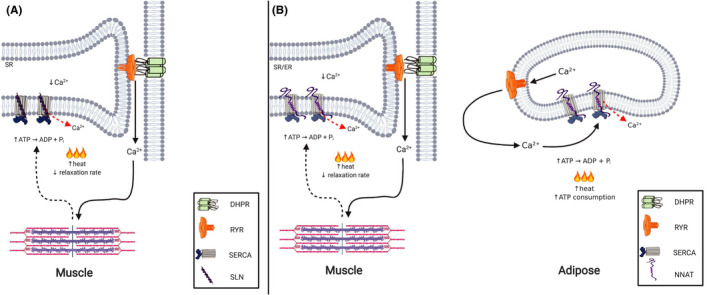
Proposed mechanism of NNAT acting as a SERCA uncoupler in skeletal muscle and adipose tissue. A, In skeletal muscle, Ca^2+^ release into the cytosol upon an action potential initiates force production in sarcomeres. SERCA acts to reduce [Ca^2+^]_i_ to elicit muscle relaxation but the presence of SLN causes Ca^2+^ slippage back out to the cytosol. The uncoupling of Ca^2+^ transport from ATP hydrolysis increases ATP hydrolysis, energy expenditure, and increased heat production. B, Based on the sequence homology of the N‐terminus of SLN and NNAT, it is proposed that NNAT may uncouple the SERCA pump in a similar mechanism in muscle. It is also proposed that NNAT uncouples SERCA in adipose tissue, similarly leading to an increase in ATP consumption and heat production

Long considered to be the functional homolog of PLN, sarcolipin (SLN) is a small 31 amino acid protein that binds to and regulates the SERCA pump.^52^, ^53^ Like PLN, SLN can reduce SERCA’s affinity for Ca^2+^. However, SLN also has the unique capability of uncoupling the SERCA pump by inducing Ca^2+^ slippage thereby increasing SERCA energy expenditure and heat release.^44^, ^45^, ^52^, ^53^ As a mediator of non‐shivering muscle‐based thermogenesis, SLN has become a “hot” topic with studies showing that its genetic deletion in mice lowers whole‐body energy expenditure rendering them more susceptible to diet‐induced obesity.^44^ These findings are similar to that reported with *Nnat* ^KO^ mice,^30^ and interestingly, our sequence alignment analyses show that NNAT also shares sequence homology with SLN (Figure 2B). Specifically, there is significant homology in its transmembrane domain, that is predicted to bind to SERCA. That is, the transmembrane domains of both SLN and PLN share a common binding site in between SERCA’s M2, M4, M6, and M9 transmembrane helices.^54^, ^55^ Based on sequence homology, it is possible that NNAT would also fit into this groove, however, this should be specifically examined in the future. Furthermore, it is interesting to note that NNAT also shares sequence homology with the N‐terminal cytoplasmic domain of SLN. This is important as previous work has shown that SLN’s uncoupling action is dependent on the presence of its N‐terminal domain, where in fact, PLN mutants harboring SLN’s N‐terminal domain gain the SERCA uncoupling function,^56^ This raises the possibility, that like SLN, NNAT may function as a SERCA uncoupler and mediator of muscle‐based thermogenesis. In our hands, Western blot analyses detects NNAT at approximately 13 kDa (Figure 3A); and we find that like SLN, NNAT protein can be found in murine skeletal muscle (Figure 3B). However, unlike SLN, NNAT can be found in both slow type soleus and fast type extensor digitorum longus (EDL) muscles, albeit to a greater extent in the soleus (Figure 3B). Co‐immunoprecipitation experiments in the soleus also show that NNAT is capable of binding to SERCA1a and SERCA2a isoforms in skeletal muscle (Figure 3C). Even in adipose tissue where NNAT is known to be expressed, NNAT may also act to uncouple the SERCA pump. Though UCP‐1 remains the prevailing mechanism for adipose‐based thermogenesis, in the absence of UCP‐1, SERCA‐mediated futile Ca^2+^ cycling is enhanced as a compensatory mechanism.^57^, ^58^ Thus, we hypothesize that NNAT may uncouple SERCA in muscle and brown and beige adipose tissue providing alternative regulators of both muscle‐based and adipose‐based thermogenesis (Figure 4). Should NNAT prove to be a SERCA uncoupler, it would provide an avenue to increasing whole‐body energy expenditure and may be used a therapeutic target for obesity and type 2 diabetes.

## 7 CONCLUSION

The global prevalence of obesity and type 2 diabetes is increasing at alarming rates placing impetus on the discovery of novel interventions.^18^, ^59^ In this article, we have discussed the role of NNAT in glucose homeostasis, appetite regulation, adipocyte function, and energy homeostasis, making NNAT a viable therapeutic target in combatting obesity and glucose intolerance. Adding to this, we hypothesize that NNAT may mediate muscle‐ and/or adipose‐based thermogenesis via SERCA uncoupling. Only further investigation into NNAT acting as a whole‐body metabolic regulator will reveal its true importance.

## CONFLICT OF INTEREST

The authors declare no conflict of interest.

## AUTHOR CONTRIBUTIONS

J.L. Braun and V.A. Fajardo designed proposed research and article. J.L. Braun performed research. J.L. Braun, M.S. Geromella, S.I. Hamstra, and V.A. Fajardo wrote the paper.

8
